# Data and meta-analysis for choosing sugammadex or neostigmine for routine reversal of rocuronium block in adult patients

**DOI:** 10.1016/j.dib.2020.106241

**Published:** 2020-08-30

**Authors:** William E. Hurford, Mark H. Eckman, Jeffrey A. Welge

**Affiliations:** a Department of Anesthesiology, University of Cincinnati, PO Box 670531, Cincinnati, OH, United States; b Department of Medicine, University of Cincinnati, Cincinnati, OH, United States; c Department of Psychiatry and Behavioral Neuroscience, University of Cincinnati, Cincinnati, OH, United States

**Keywords:** Adult, Gamma-cyclodextrins, Meta-analysis, Neostigmine, Neuromuscular blocking agents, Sugammadex

## Abstract

This meta-analysis was conducted to define clinical efficacy and side effects (bradycardia and post-operative nausea and vomiting [PONV]) in trials comparing sugammadex with neostigmine or placebo for reversal of rocuronium-induced neuromuscular blockade in adult patients. A search of PubMed, Google Scholar, and Cochrane Library electronic databases identified 111 clinical trials for potential inclusion. We performed a meta-analysis of 32 studies that quantitatively compared the efficacy and side effects of sugammadex with either neostigmine or placebo in adult patients requiring general anesthesia. Analyzed outcomes were reversal time, anesthesia time, duration of stay in the post-anesthesia care unit (PACU), and the occurrence of bradycardia or PONV. Odds ratios and 95% confidence intervals (CI) were calculated for binary data. Mean differences and 95% CI were calculated for continuous outcome data. Meta-analyses were performed using random and fixed-effects models. Heterogeneity across studies was assessed using Cochran's Q test and the I^2^ statistic. Quantification of these outcomes can better inform anesthetists and health systems of the relative costs and benefits of the two reversal agents. This information also forms a basis for a comparative cost analysis in a co-submitted manuscript [1].

## Specifications Table

**Table:** 

Subject	Anesthesiology and Pain Medicine
Specific subject area	Reversal of rocuronium neuromuscular blockade with sugammadex or neostigmine.
Type of data	Table Chart Figure
How data were acquired	Systematic review and meta-analysis
Data format	Raw Analyzed
Parameters for data collection	The primary outcomes recorded were: time to recovery of the train-of-four ratio to > 0.9; total anesthesia time; time from admission to the post-anesthesia recovery unit (PACU) until the patient was ready for discharge from the unit; occurrence of bradycardia; occurrence of post-operative nausea and vomiting (PONV).
Description of data collection	A search of PubMed, Google Scholar, and Cochrane Library electronic databases identified 111 clinical trials for potential inclusion. We performed screening of citations, data extraction, and quality assessment in duplicate. We performed a meta-analysis of 32 studies that quantitatively compared the efficacy and side effects of sugammadex with either neostigmine or placebo in adult patients requiring general anesthesia.
Data source location	University of Cincinnati Cincinnati, Ohio United States
Data accessibility	With the article
Related research article	Hurford WE, Welge JA, Eckman MH. Sugammadex versus neostigmine for routine reversal of rocuronium block in adult patients: A cost analysis. J. Clin. Anesth. In Press. https://doi.org/10.1016/j.jclinane.2020.110027.

## Value of the Data

• This meta-analytic data quantifies and updates our current level of understanding of the comparative efficacy and side effects of a newer, more expensive reversal drug, sugammadex, with its generic counterpart, neostigmine.
• Quantification of these outcomes can inform anesthetists and health systems of the relative costs and benefits of the two reversal agents.
• This information provides a basis for undertaking comparative cost analyses that can inform clinical and administrative decisions within hospitals and health systems.

## 1 Data Description

### 1.1 Study flow and description of data

**Fig. 1:**
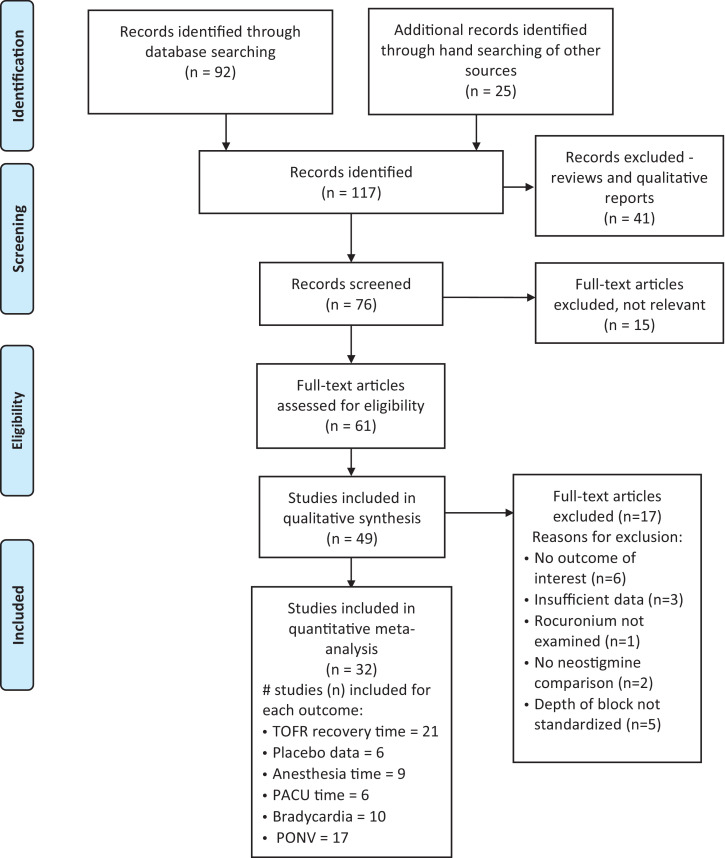
PRISMA Diagram.

The initial literature search identified 117 reports for potential inclusion (Fig. 1). Forty-one of these reports were reviews or qualitative descriptions. The remaining 76 reports were screened; 15 were excluded as not being relevant. Of the 61 studies assessed for eligibility, 17 did not meet inclusion criteria and were excluded. The remaining 32 reports were submitted to quantitative meta-analysis. The characteristics of the included studies are listed in Table 1. Table 2 lists the 17 excluded studies and the reasons for exclusion. All data are provided in the attached supplemental Excel file.

**Table 1:** Characteristics of Included Studies.

Study	Title	Journal	Study Design	# Subjects	Block Depth	Special Population	Sugammadex Dose	Neostigmine Dose	Comments
Adamus, 2011	Intraoperative reversal of neuromuscular block with sugammadex or neostigmine during extreme lateral interbody fusion, a novel technique for spine surgery	J Anes 2011; 25:716–20	Single center randomized trial	22	Moderate	Yes, spine surgery	2 mg/kg	40 mcg/kg	
Blobner, 2010	Reversal of rocuronium-induced neuromuscular blockade with sugammadex compared with neostigmine during sevoflurane anesthesia: results of a randomised controlled trial	Eur J Anaesthesiol 2010; 27:874–81	Multicenter randomized trial	98	Moderate	No	2 mg/kg	50 mcg/kg	
Brueckmann, 2015	Effects of sugammadex on incidence of postoperative residual neuromuscular blockade: a randomized, controlled study	Br J Anaesth 2015; 155:743–51	Single center randomized tiral	154	Deep	No	2 or 4 mg/kg	50 mcg/kg	
Carron, 2013	Sugammadex Allows Fast-Track Bariatric Surgery	Obes Surg 2013; 23:1558–1563	Single center randomized trial	40	Deep	Yes, obesity	4 mg/kg	60 mcg/kg	
Carron, 2016	Sugammadex for reversal of neuromuscular blockade: a retrospective analysis of clinical outcomes and cost-effectiveness in a single center	ClinicoEconomics and Outcomes Research 2016: 8 43–52	Single center retrospective matched cohort study	101	Various	No	Various	Various	
Castro, 2014	Sugammadex reduces postoperative pain after laparoscopic bariatric surgery: a randomized trial	Surg Laparosc Endosc Percutan Tech 2014; 24:420–423	Single center randomized trial	88	Moderate	Yes, obesity	2 mg/kg	50 mcg/kg	
Cheong, 2015	The combination of sugammadex and neostigmine can reduce the dosage of sugammadex during recovery from the moderate neuromuscular blockade	Korean J Anesthesiol 2015; 68:547–555	Single center randomized trial	60	Moderate	No	2 mg/kg	50 mcg/kg	
De Robertis, 2016	The use of sugammadex for bariatric surgery: analysis of recovery time from neuromuscular blockade and possible economic impact	ClinicoEconomics and Outcomes Research 2016; 8:317–22	Single center randomized trial	99	Moderate	Yes, obesity	2 mg/kg	50 mcg/kg	
El Sherbeny, 2017	Efficacy and safety of sugammadex in reversing nmb (rocuronium) in adults	New York Science Journal 2017; 10: 22–29	Single center randomized trial	40	Shallow	No	3 mg/kg	50 mcg/kg	
Gaszynski, 2012	Randomized comparison of sugammadex and neostigmine for reversal of rocuronium-induced muscle relaxation in morbidly obese undergoing general anesthesia	Br J Anaesth 2012; 108:236–9	Single center randomized trial	70	Moderate	Yes, obesity	2 mg/kg	50 mcg/kg	
Georgiou, 2013	Clinical and cost-effectiveness of sugammadex versus neostigmine reversal of rocuronium-induced neuromuscular block in super obese patients undergoing open laparotomy for bariatric surgery. A randomized controlled trial: 9AP1–7	Eur J Anaesthesiol 2013: 30:141 (abstract)	Single center randomized trial	29	Moderate	Yes, obesity	2 mg/kg	50 mcg./kg	
Grintescu, 2009	Comparison of the cost-effectiveness of sugammadex and neostigmine during general anesthesia for laparoscopic cholecystectomy	Br J Anaesth 2009; 103: 917p (abstract)	Single center open randomized trial	34	Moderate	No	2 mg/kg	50 mcg/kg	
Illman, 2011	The duration of residual neuromuscular block after administration of neostigmine or sugammadex at two visible twitches during train-of-four monitoring	Anesth Analg 2011; 112:63–8	Single center double-blind randomized trial	27	Moderate	No	2 mg/kg	50 mcg/kg	
Jones, 2008	Reversal of profound rocuronium-induced blockade with sugammadex: a randomized comparison with neostigmine	Anesthesiology 2008; 109:816–24	Multicenter randomized trial	75	Deep	No	4 mg/kg	70 mcg/kg	
Kaufhold, 2016	Sugammadex and neostigmine dose-finding study for reversal of residual neuromuscular block at a train-of-four ratio of 0.2 (SUNDRO20)	Br J Anaesth. 2016 ; 116:233–40	Single center double-blind randomized dose-finding trial	18	Shallow	No	1.25 mg/kg	55 mcg/kg	Placebo data (*n* = 9)
Koc, 2015	Comparison of sugammadex and neostigmine for reversal of rocuronium-induced muscle relaxation in short term elective surgery	J Clin Analytical Med 2015; 6:41–4	Single center randomized trial	33	Moderate	Yes, brief surgery	2 mg/kg	50 mcg/kg	
Kogler, 2012	Sugammadex reversal of rocuronium-induced neuromuscular block in interventional bronchoscopy procedures: a comparison with neostigmine	Eur J Anaesth 2012; 29:146 (abstract)	Single center randomized trial	31	Deep	Yes, bronchoscopy	2 mg/kg	70 mcg/kg	
Koyuncu, 2015	Comparison of sugammadex and conventional reversal on postoperative nausea and vomiting: a randomized, blinded trial	Journal of Clinical Anesthesia 2015; 27: 51–56	Single center randomized trial	100	Shallow	No	2 mg/kg	70 mcg/kg	
Mekawy, 2012	Improved recovery profiles in sinonasal surgery. Sugammadex: Does it have a role?	Egyptian Journal of Anaesthesia 2012; 28:175–178	Single center randomized trial	40	Moderate	Yes, sinus surgery	4 mg/kg	50 mcg/kg	
Paech, 2018	recovery characteristics of patients receiving either sugammadex or neostigmine and glycopyrrolate for reversal of neuromuscular block: a randomised controlled trial	Anesthesia 2018; 73:340–347	Single center randomized trial	304	Moderate	Yes, women undergoing elective day-surgical laparoscopic gynecologic surgery	2 mg/kg	40 mcg/kg	
Pongracz, 2013	Reversal of neuromuscular blockade with sugammadex at the reappearance of four twitches to train-of-four stimulation	Anesthesiology 2013; 119:36–42.	Double-blind randomized single center study	36	Shallow	No	2 mg/kg	50 mcg/kg	
Puhringer, 2010	Sugammadex rapidly reverses moderate rocuronium- or vecuronium-induced neuromuscular block during sevoflurane anesthesia: a dose–response relationship	Br J Anaesthesia 2010; 105 610–19	Single center randomized trial	19	Moderate	No	2 mg/kg	na	Placebo data (*n* = 10)
Rahe-Meyer, 2015	Recovery from prolonged deep rocuronium-induced neuromuscular blockade A randomized comparison of sugammadex reversal with spontaneous recovery	Anaesthesist 2015; 64:506–512	Multicenter randomized trial	134	Deep	No	4 mg/kg	na	Placebo data (*n* = 65)
Sabo, 2011	Residual neuromuscular blockade at extubation: a randomized comparison of sugammadex and neostigmine reversal of rocuronium-induced blockade in patients undergoing abdominal surgery	J Anesthe Clinic Res 2011; 2:140	Multicenter randomized trial	100	Deep	No	4 mg/kg	50 mcg/kg	
Sacan, 2007	Sugammadex reversal of rocuronium-induced neuromuscular blockade: a comparison with neostigmine–glycopyrrolate and edrophonium–atropine	Anesth Analg 2007; 104:569 –74	Single center, open-label prospective trial	40	Moderate	No	4 mg/kg	70 mg/kg	
Schaller, 2010	Sugammadex and neostigmine dose-finding study for reversal of shallow residual neuromuscular block	Anesthesiology 2010; 113:1054 – 60	Single center randomized double-blind trial	96	Shallow	No	1 mg/kg	40 mcg/kg	Placebo data (*n* = 9)
Sorgenfrei, 2006	Reversal of rocuronium-induced neuromuscular block by the selective relaxant binding agent sugammadex. a dose-finding and safety study	Anesthesiology 2006; 104:667–74	Multicenter dose finding study with placebo	27	Moderate	No	2 mg/kg	na	Placebo data (*n* = 4)
Sparr, 2007	Early reversal of profound rocuronium-induced neuromuscular blockade by sugammadex in a randomized multicenter study	Anesthesiology 2007; 106:935– 43	Multi-center dose-finding study; Phase II trial	98	Shallow	No	2 mg/kg	na	Placebo data (*n* = 6)
Unal, 2015	Comparison of sugammadex versus neostigmine costs and respiratory complications in patients with obstructive sleep apnea	Turk J Anaesth Reanim 2015; 43:387–95	Single center randomized trial	74	Moderate	Yes, sleep apnea	2 mg/kg	40 mcg/kg	
Woo, 2013	Sugammadex versus neostigmine reversal of moderate rocuronium-induced neuromuscular blockade in Korean patients	Korean J Anesthesiol 2013; 65:501–507	Multicenter randomized trial	118	Moderate	No	2 mg/kg	50 mcg/kg	
Wu, 2014	Rocuronium blockade reversal with sugammadex vs. neostigmine: randomized study in Chinese and Caucasian subjects	BMC Anesthesiology 2014 Jul12; 14:53	Multicenter randomized trial	291	Moderate	No	2 mg/kg	50 mcg/kg	
Yagan, 2015	Intraocular pressure changes associated with tracheal extubation: Comparison of sugammadex with conventional reversal of neuromuscular blockade	J Pak Med Assoc 2015; 65:1219–25	Randomized single center trial	36	Shallow	No	2 mg/kg	50 mcg/kg

**Table 2:** Characteristics of Excluded Studies.

Study	Title	Journal	Study Design	# Subjects	Block Depth	Special Population	Sugammadex Dose	Neostigmine Dose	Comments
Amorin, 2014	Neostigmine vs. sugammadex: observational cohort study comparing the quality of recovery using the Postoperative Quality Recovery Scale	Acta Anaesthesiol Scand 2014; 58:1101–1110	Single center convenience sample	101	Various	No	na	na	Block not standardized
Balaka, 2011	Comparison of sugammadex to neostigmine reversal of neuromuscular blockade in patients with myasthenia gravis	J Cardiothorac Vasc Anes 2011; 25:S22-S23	Single center randomized trial	40	Shallow	Yes, myasthenia gravis	4 mg/kg	2.5 mg	Insufficient data
Boon, 2016	Improved postoperative oxygenation after antagonism of moderate neuromuscular block with sugammadex versus neostigmine after extubation in 'blinded' conditions.	Br J Anaesth. 2016; 117:410–1	Multicenter double blind trial	100	Moderate	No	2 mg/kg	2.5 mg	No outcomes of interest
Flockton, 2008	Reversal of rocuronium-induced neuromuscular block with sugammadex is faster than reversal of cisatracurium-induced block with neostigmine	Br J Anaesth 2008; 100:622–30	Single center randomized trial	73	Moderate	No	2 mg/kg	50 mcg/kg	Rocuronium not compared in both groups
Geldner, 2012	a randomised controlled trial comparing sugammadex and neostigmine at different depths of neuromuscular blockade in patients undergoing laparoscopic surgery	Anesthesia 2012; 67:991–998	Multicenter randomized trial	140	Various	No	4 mg/kg	50 mcg/kg	Block not standardized
Hakimoglu, 2016	Comparison of sugammadex and neostigmine-atropine on intraocular pressure and postoperative effects	Kaohsiung J Med Sci 2016; 32:80–5	Single center randomized trial	60	Moderate	No	4 mg/kg	50 mcg/kg	No outcomes of interest
Kizilay, 2016	Comparison of neostigmine and sugammadex for hemodynamic parameters in cardiac patients undergoing noncardiac surgery	J Clin Anesth 2016; 28:30–5	Single center randomized trial	90	Moderate	No	3 mg/kg	30 mcg/kg	No outcomes of interest
Ledowski, 2014	Retrospective investigation of postoperative outcome after reversal of residual neuromuscular blockade Sugammadex, neostigmine or no reversal.	Eur J Anaesthesiol 2014; 31:423–29	Single center retrospective cohort study	1444	Various	No	Various	Various	Block not standardized
Martinez-Ubieto, 2016	Prospective study of residual neuromuscular block and postoperative respiratory complications in patients reversed with neostigmine versus sugammadex	Minerva Anestesiol 2016; 82:735–42	Single center prospective cohort study	325	Various	No	Various	Various	Insufficient data
Nemes, 2017	Impact of reversal strategies on the incidence of postoperative residual paralysis after rocuronium relaxation without neuromuscular monitoring. A partially randomised placebo controlled trial	Eur J Anaesthesiol 2017; 34:609–616	Partially randomized placebo-controlled trial	125	na	No	2 mg/kg	60 mcgkg	Block not standardized
Oh, 2019 [7]	Retrospective analysis of 30-day unplanned readmission after major abdominal surgery with reversal by sugammadex or neostigmine	Br J Anaesth 2019; 122:370–378	Single center retrospective cohort study	1479	na	No	> 2 mg/kg	30 – 50 mcg/kg	No outcomes of interest
Olesnicky, 2016	The effect of routine availability of sugammadex on postoperative respiratory complications: a historical cohort study	Minerva Anestesiol 2017; 83:248-254	Single center retrospective pre-post study	922	Various	No	Various	Various	No comparison with neostigmine or placebo
Raziel, 2013	Comparison of two neuromuscular anesthesics reversal in obese patients undergoing bariatric surgery - A prospective study	Conference Paper in Obesity Surgery, Vienna, Austria. August 2013	Single center randomized trial	40	na	Yes, obesity	na	na	Insufficient data
Sauer, 2011	The influence of residual neuromuscular block on the incidence of critical respiratory events. A randomised, prospective, placebo-controlled trial	Eur J Anaesthesiol 2011; 28:842–848	Single center randomized trial	132	Deep	No	na	20 mcg/kg vs. placebo	No outcomes of interest
Sherman, 2014	The effect of sugammadex vs. neostigmine on the postoperative respiratory complications following laparoscopic sleeve gastrectomy	Eur J Anaesthesiol 2014; Abstract 9AP4–5	Single center randomized trial	57	Various	Yes, obesity	2 mg/kg	2.5 mg	No outcomes of interest
Stourac, 2016	Low-dose or high-dose rocuronium reversed with neostigmine or sugammadex for cesarean delivery anesthesia: a randomized controlled noninferiority trial of time to tracheal intubation and extubation	Anesth Analg 2016; 122:1536–45	Two center randomized trial	240	Various	Yes, parturients	2 – 4 mg/kg	30 mcg/kg	Block not comparable in both groups
Watts, 2012	The influence of unrestricted use of sugammadex on clinical anesthetic practice in a tertiary teaching hospital	Anaesth Intensive Care 2012; 40: 333–339	Single center retrospective case audit	374	Various	No	Various	Various	No comparison with neostigmine or placebo

**Fig. 2:**
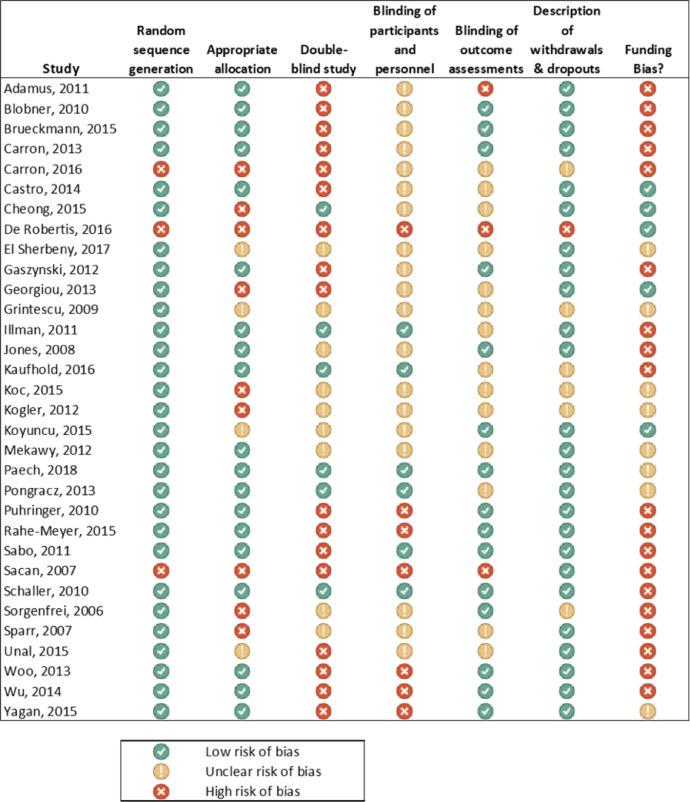
Risk of bias summary for studies included in the meta-analysis.

The assessment of possible study bias in the included studies is outlined in Fig. 2. Two of the reports studied two distinct subject samples. Sparr et al. separated their dose-finding study into subjects with either a deep (*n* = 6) or shallow (*n* = 9) levels of neuromuscular blockade prior to reversal [2]. Woo and colleagues reported two separate subject samples: a caucasian group (*n* = 59) and a Chinese group (*n* = 130) [3]. In both studies, the groups were analyzed separately since merged data were not available in the original articles.

### 1.2 Train-of-four recovery (TOFR)

**Fig. 3:**
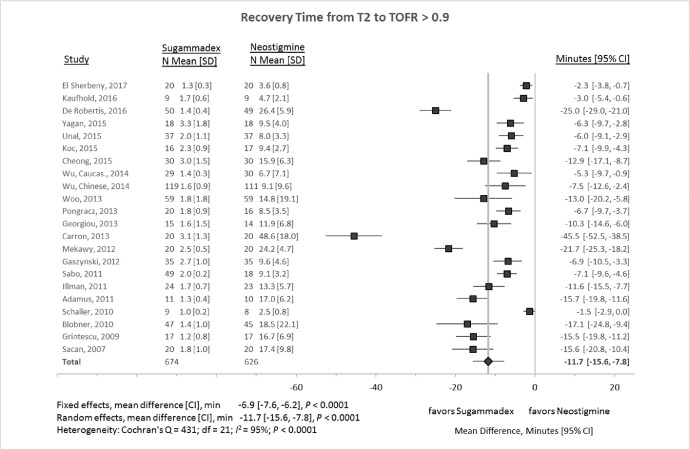
Recovery times (from two of four twitches (T2) in a train-of-four to ≥ 0.9 recovery of twitch height) after administration of sugammadex or neostigmine. CI, 95% confidence interval; N, number of subjects; SD, standard deviation.

Fig. 3 outlines a meta-analysis of 22 studies that quantified the mean difference in train-of-four (TOF) recovery time to at least 90% of complete reversal. The mean difference between therapies was 11.7 min (95% confidence interval [CI] −15.6 to −7.8 min, *P* < 0.0001; *I*^2^ = 95%). Sensitivity analyses, omitting each study in turn, produced random-effect mean differences ranging between −10.1 to −12.2 min (*P* < 0.0001 for all analyses). Two studies, Carron et al. and DeRobertis et al., visually appeared to be outliers [4,5]. These reports were also the only two retrospective studies included in the analysis. Omitting both these studies in a sensitivity analysis resulted in a random-effects mean difference of −9.3 min (95% CI −11.8 to −6.9 min, *P* < 0.0001; *I*^2^ = 92%).

**Fig. 4:**
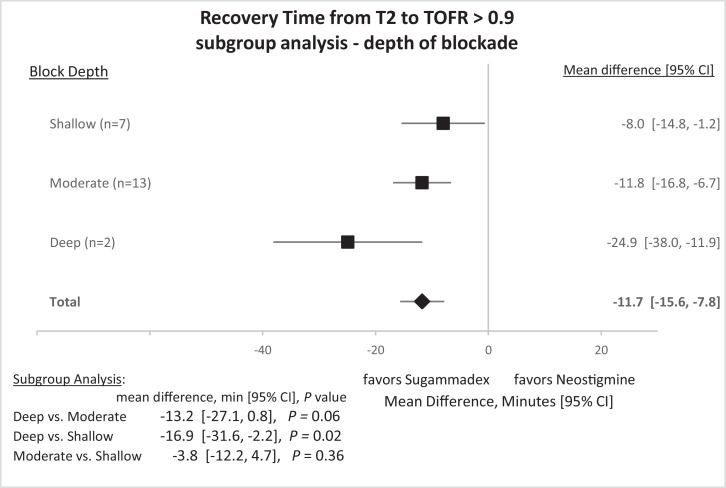
Recovery times from two of four twitches (T2) in a train-of-four (TOF) to ≥ 0.9 recovery of twitch height after administration of sugammadex or neostigmine. Subgroup analysis by depth of neuromuscular block prior to reversal: shallow block (4 of 4 twitches present in TOF), moderate block (2 of 4 twitches present in TOF), deep blockade (1 to 2 twitches of TOF present after post-tetanic stimulation). CI, 95% confidence interval.

Subgroup analyses were conducted on the depth of blockade (Fig. 4). The mean difference for reversing deep blockade (1 to 2 twitches of TOF present after post-tetanic stimulation; *n* = 2 studies) [4,6] was −24.9 min (95% CI −38.0 to −11.9 min, *P* = 0.0008), for moderate block (2 of 4 twitches present in TOF; *n* = 13 studies) −11.8 min (95% CI −16.8 to −6.7 min, *P* = 0.0001), and for shallow block (4 of 4 twitches present in TOF; *n* = 7 studies) was −8.0 min (95% CI −14.8 to −1.2 min, *P* = 0.023).

### 1.3 Train-of-four recovery (TOFR) – sugammadex compared to placebo

**Fig. 5:**
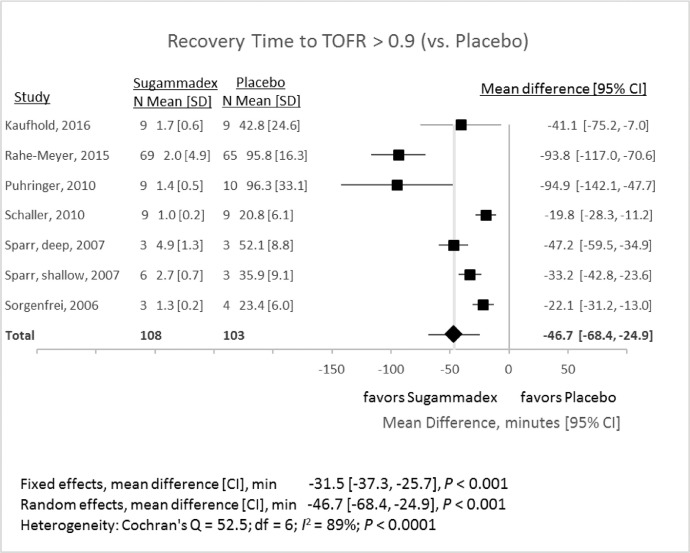
Recovery times (from two of four twitches (T2) in a train-of-four to ≥ 0.9 recovery of twitch height) after administration of sugammadex or placebo. CI, 95% confidence interval; SD, standard deviation.

Six studies compared reversal with sugammadex to a placebo (Fig. 5). One study (Sparr, 2007) compared reversal of TOF at two different levels of block prior to reversal [2]. These groups were analyzed separately in the meta-analysis. The mean difference in reversal time between sugammadex and placebo was −46.7 min (95% CI −68.4 to −24.9 min, *P* < 0.0001; *I*^2^ = 89%). Sensitivity analyses, omitting each study in turn, produced random-effects mean differences ranging between −35.2 and −51.9 min (*P* ranging from < 0.0001 to 0.0015 for all analyses).

### 1.4 Anesthesia time

**Fig. 6:**
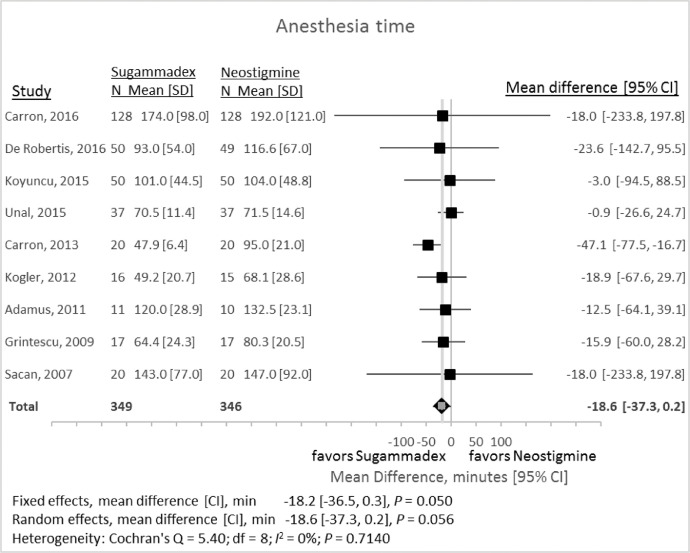
Total anesthesia time associated with reversal with either sugammadex or neostigmine. CI, 95% confidence interval; SD, standard deviation.

Anesthesia time represented the time in minutes between induction of anesthesia and extubation. Nine studies provided adequate data on anesthesia time (Fig. 6). Reversal with sugammadex compared with neostigmine resulted in a random-effects mean difference of −18.6 min (95% CI −37.3 to +0.2 min, *P* = 0.056).

### 1.5 Post-anesthesia recovery unit (PACU) time

**Fig. 7:**
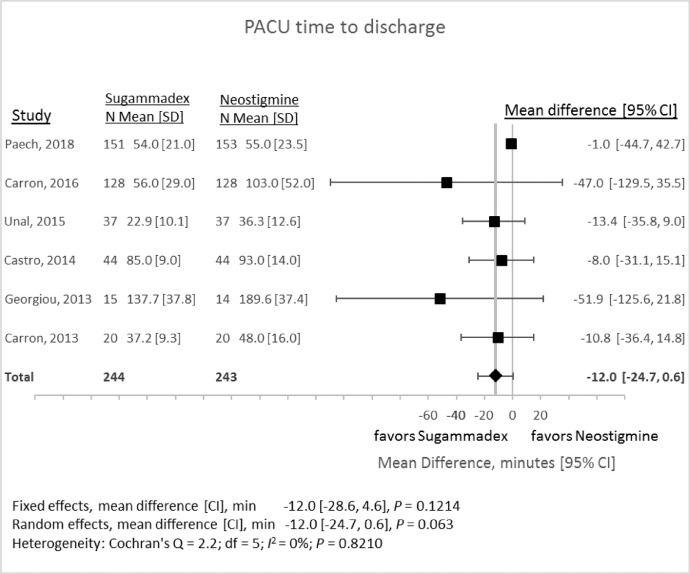
Time from admission to the Post-anesthesia Care Unit (PACU) until ready for discharge associated with reversal with either sugammadex or neostigmine. CI, 95% confidence interval; SD, standard deviation.

PACU time represented the time in minutes between a patient's admission to the PACU and the time that the patient was deemed ready for discharge. Six studies met inclusion criteria for this outcome measure (Fig. 7). Reversal with sugammadex compared with neostigmine resulted in random-effects mean difference of −12.0 min (95% CI −24.7 to +0.6 min, *P* = 0.063).

### 1.6 Occurrence of bradycardia

**Fig. 8:**
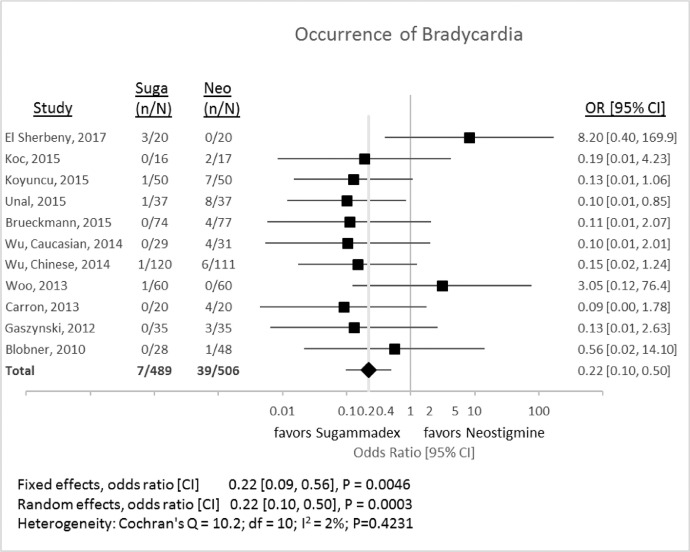
Incidence of bradycardia associated with reversal with either sugammadex (Suga) or neostigmine (Neo). CI, 95% confidence interval; Neo, neostigmine; n, number of events; N, number of subjects; OR, odds ratio; Suga, sugammadex.

Fig. 8 shows the difference in the occurrence of bradycardia, as defined by the investigators, after either sugammadex or neostigmine administration. The random-effects odds ratio was 0.22 (95% CI 0.10 to 0.50, *P* = 0.0003) for the comparison between sugammadex and neostigmine.

### 1.7 Occurrence of postoperative nausea and vomiting (PONV)

**Fig. 9:**
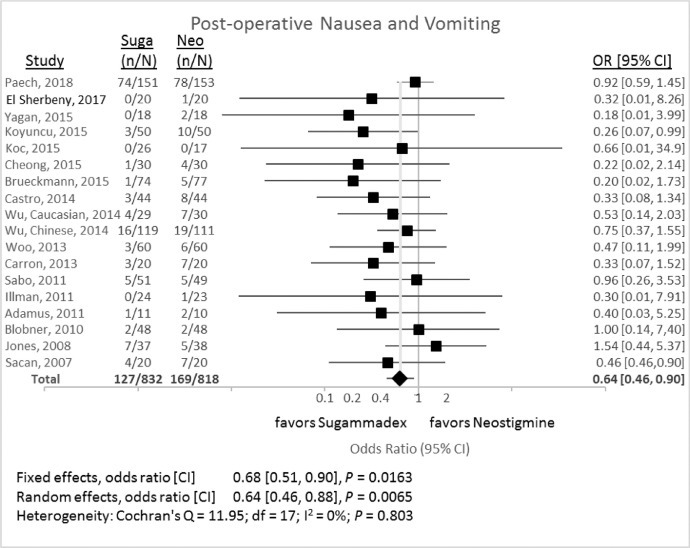
Incidence of post-operative nausea and vomiting associated with reversal with either sugammadex (Suga) or neostigmine (Neo). CI, 95% confidence interval; Neo, neostigmine; n, number of events; N, number of subjects; OR, odds ratio; Suga, sugammadex.

Fig. 9 outlines the difference in the occurrence of PONV, as defined by the investigators, after either sugammadex or neostigmine administration. The random-effects odds ratio was 0.64 (95% CI 0.46 to 0.87, *P* = 0.0065) for the comparison between sugammadex and neostigmine.

## 2 Experimental design, materials and methods

### 2.1 Search strategy

PubMed, Google Scholar, and the Cochrane library electronic databases were searched for articles published between January 1, 2005 (the publication year of the first description of sugammadex [8]) and June 1, 2019. Using the AND function, the search terms “sugammadex” OR “srba” OR “selective relaxant binding agent” were combined with “neostigmine OR placebo” and “rocuronium.” The search population then was limited to “human,” and “adult.” Titles and abstracts for all articles returned by the search strategy were screened. The reference lists of each article, as well as previously-published reviews and meta-analyses, were manually searched for additional references of potential interest. The full texts of each article were then retrieved to assess suitability for inclusion. This manuscript adheres to applicable PRISMA (Preferred Reporting Items for Systematic Reviews and Meta-Analyses) guidelines (http://www.prisma-statement.org, accessed May 15, 2020).

### 2.2 Inclusion/exclusion criteria

We identified randomized clinical trials and cohort studies with the following inclusion criteria: sugammadex, at a dose between 1 mg/kg and 4 mg/kg, was used for reversal of rocuronium-induced neuromuscular blockade; the effects of sugammadex were directly compared with either neostigmine, at a dose of 30 to 80 mcg/kg, or placebo; the depth of neuromucular blockade was objectively quantified and was similar in each group; patients were at least 18 years of age and undergoing a procedure requiring general anesthesia; adequate data were present either in English or an understandable graphic format. Exclusion criteria included: pediatric studies; case series or pre-post time series studies; inability to retrieve a full-text version or English abstract; lack of outcome data of interest.

### 2.3 Quality assessment

We assessed the study design of articles. Both cohort and randomized studies were included. Assessment for potential bias was performed according PRISMA methodology. No studies were excluded for a specific level of potential bias.

### 2.4 Data extraction

Data were extracted from the original texts and summarized in an Excel database. We performed screening of citations, data extraction, and quality assessment in duplicate. Prior to finalization, we verified the database against the original reports and corrected as necessary. The primary outcomes recorded were: time to recovery of the train-of-four ratio to ≥ 0.9; total anesthesia time; time from admission to the post-anesthesia recovery unit (PACU) until the patient was ready for discharge from the unit; occurrence of bradycardia, as defined by the investigators; occurrence of post-operative nausea and vomiting (PONV), as defined by the investigators.

Studies were categorized as to patient population (general surgical versus special population [study sample limited to a specific high-risk procedure or patient population]) and depth of neuromuscular blockade (deep – post-tetanic facilitation only; moderate – return of two of four twitches in a train-of four [TOF] stimulus; or shallow – return of a TOF ratio of 0.1 to 0.9).

### 2.5 Statistical methods

We calculated odds ratios (OR) and 95% confidence intervals (CI) for binary data. Mean difference (MD) and 95% CI were calculated for continuous outcome data. Time-based data in which median and ranges were reported were converted to estimate means and standard deviations according to the techniques outlined by Hozo et al. [9]

Meta-analysis was performed using random- and fixed-effects models. The random-effects model appeared more appropriate since it was expected that variation among studies would occur beyond that associated with sampling variation. When calculating odds ratios, 0.5 was added to the frequencies of each cell in studies with a zero number of events within a cell. Heterogeneity across studies was assessed using Cochran's Q test and the *I*^2^ statistic. The *I*^2^ statistic estimated the percentage of total variation among the study effects attributable to heterogeneity among studies rather than sampling error.

All *P* values were two-tailed, and a *P* value < 0.05 was considered to represent statistical significance. Computations were performed with SAS Enterprise Guide Version 7.15 (SAS Institute Inc., Cary, NC) using PROC MIXED. Data are reported as forest plots for each outcome. Point estimates and 95% confidence intervals (CI) are reported for individual studies. Mean differences for continuous variables and mean odd ratios for dichotomous variables, along with 95% CI are reported for fixed and random-effects models.

## 3 Ethics statement

Our Institutional Review Board determined that this analysis did not meet the regulatory criteria for research involving human subjects.

## Declaration of Competing Interest

The authors declare that they have no known competing financial interests or personal relationships which have, or could be perceived to have, influenced the work reported in this article.

## Appendix Supplementary materials

Image, application 1
